# Supplementary material for: Oxygen Gradient Induced in Microfluidic Chips Can Be Used as a Model for Liver Zonation
Source: Cells. 2022 Nov 23;11(23):3734. doi: 10.3390/cells11233734 (PMC9738923; doi:10.3390/cells11233734)
Supplement: Supplementary file 1 [file cells-11-03734-s001.zip › cells-1750950-SM.pdf]

cells

MDPI

Article

Oxygen gradient induced in microfluidic chips can be used as a model for liver zonation

Shahrouz Ghafoory[1,†], Christina Steng[2,3,4,†], Stefan Kopany[1], Mert Mayadag[1], Nils Mechtel[1], Brennah Murphy[5] Sebastian Schattschneider[6], Niklas Wilhelmi[6] & Stefan Wölfl[1*]

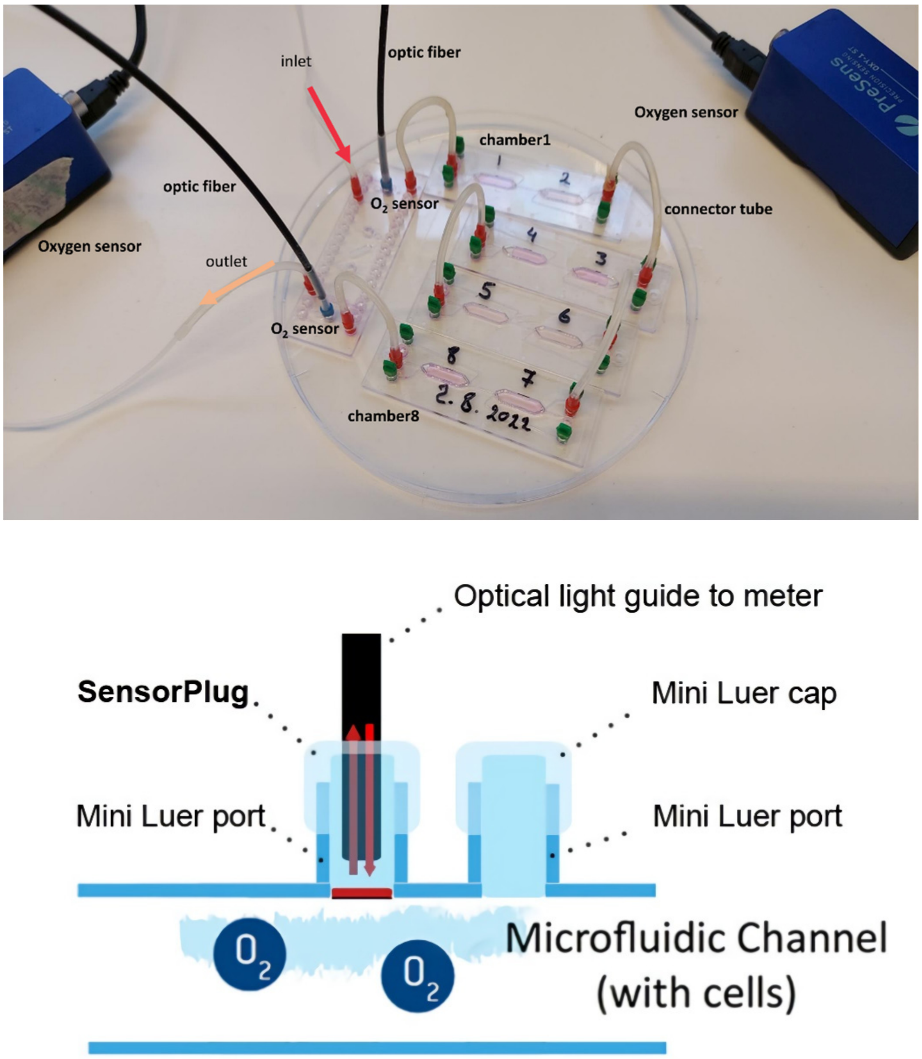

Supplementary Figure S1: Oxygen sensor for O_2 measurement in MCs. The inlet is connected to the individually manufactured MC (lot number: 1090) in which an optic fiber is placed to measure oxygen before the MCs. Next, media flows from chamber1 to chamber8. After the last MC (chamber8) another oxygen sensor is positioned to measure oxygen in the media after it is flown through the MCs (A). Schematical drawing of the oxygen measurement with oxygen sensor in the media (B).

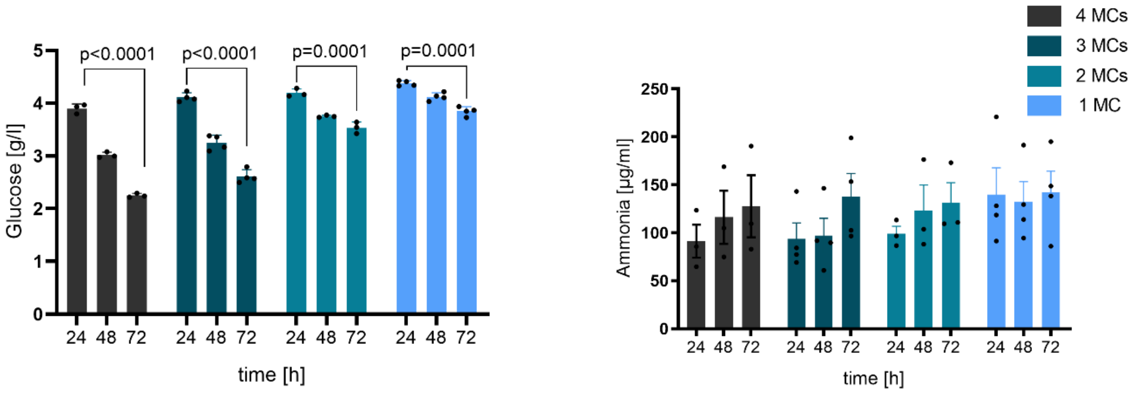

Supplementary Figure S2: Glucose and ammonia concentration in MCs 1-4. In each of the four conditions (1, 2, 3 and 4 connected MCs) a significant difference in glucose concentration between 24h and 72h was found (A). For ammonia, no significant difference in any of the shown data was found. Comparing each ammonia concentration at 72h revealed a p>0.99.

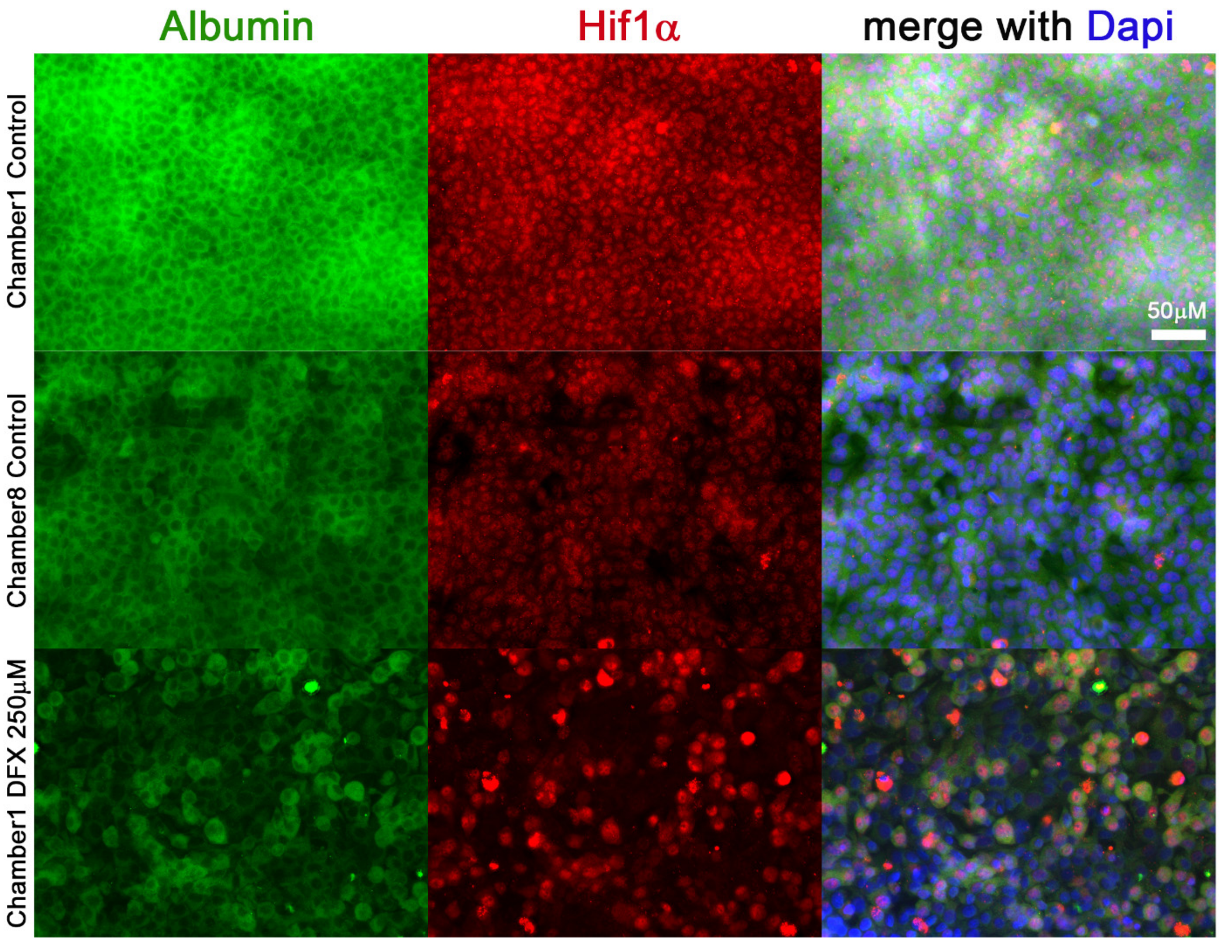

Supplementary Figure S3: Albumin and Hif1α immunofluorescence co-staining for chamber1, chamber8 and chamber1 treated with DFX. IF staining of albumin (green) and Hif1α (red) in chamber1, chamber8 (normal media) and chamber1 treated with 250µM DFX. Scale bar 50µM. A merge with the DAPI image is shown in the right column.

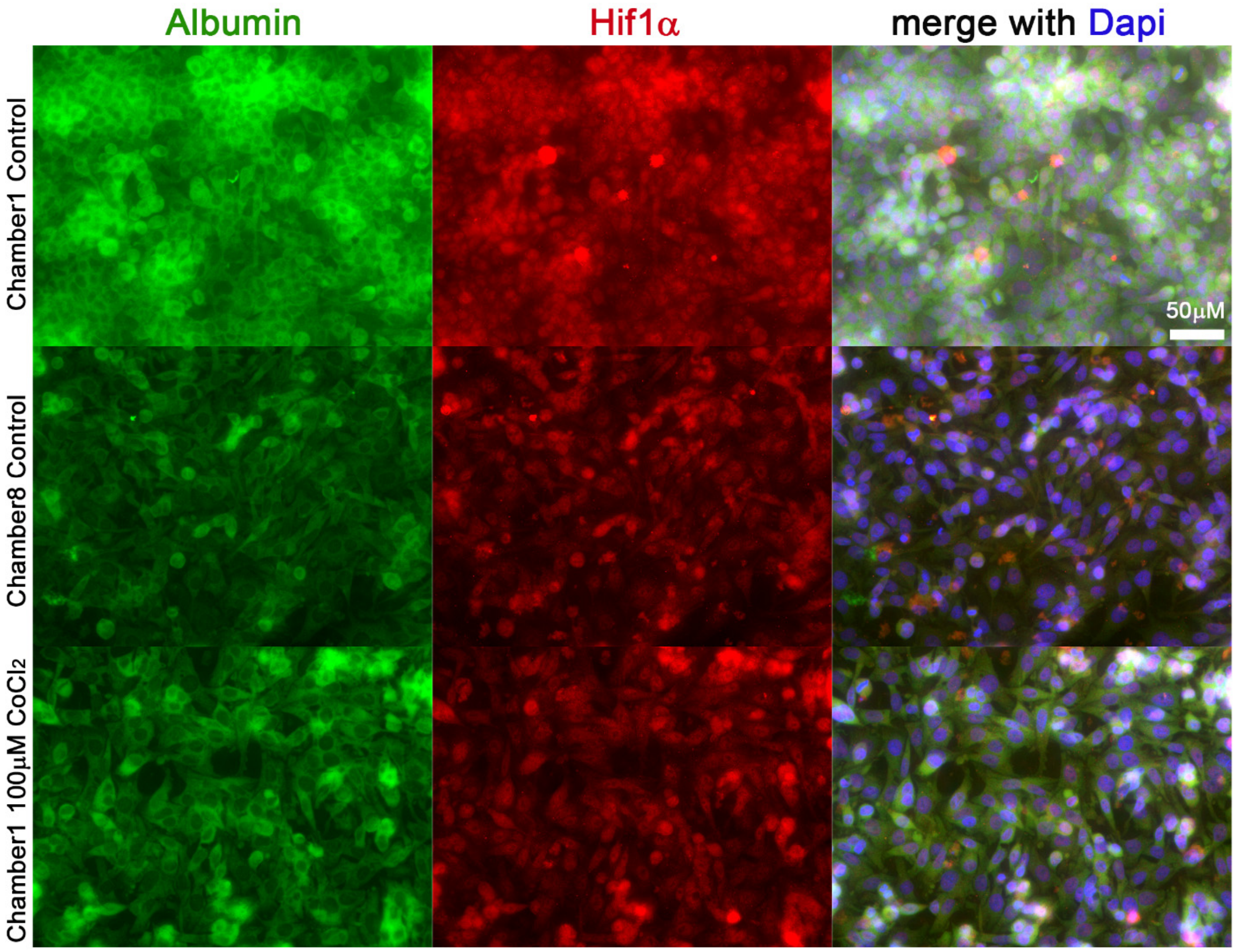

Supplementary Figure S4: IF staining of albumin and Hif1α in chamber1, chamber8 (normal media) and chamber1 treated with $CoCl_2$. IF staining of HepG2 cells after 72h for chamber1, chamber8 (normal media) and chamber1 treated with 100µM $CoCl_2$. Albumin is visualized in green and Hif1α is shown in red. Scale bar 50 µm.

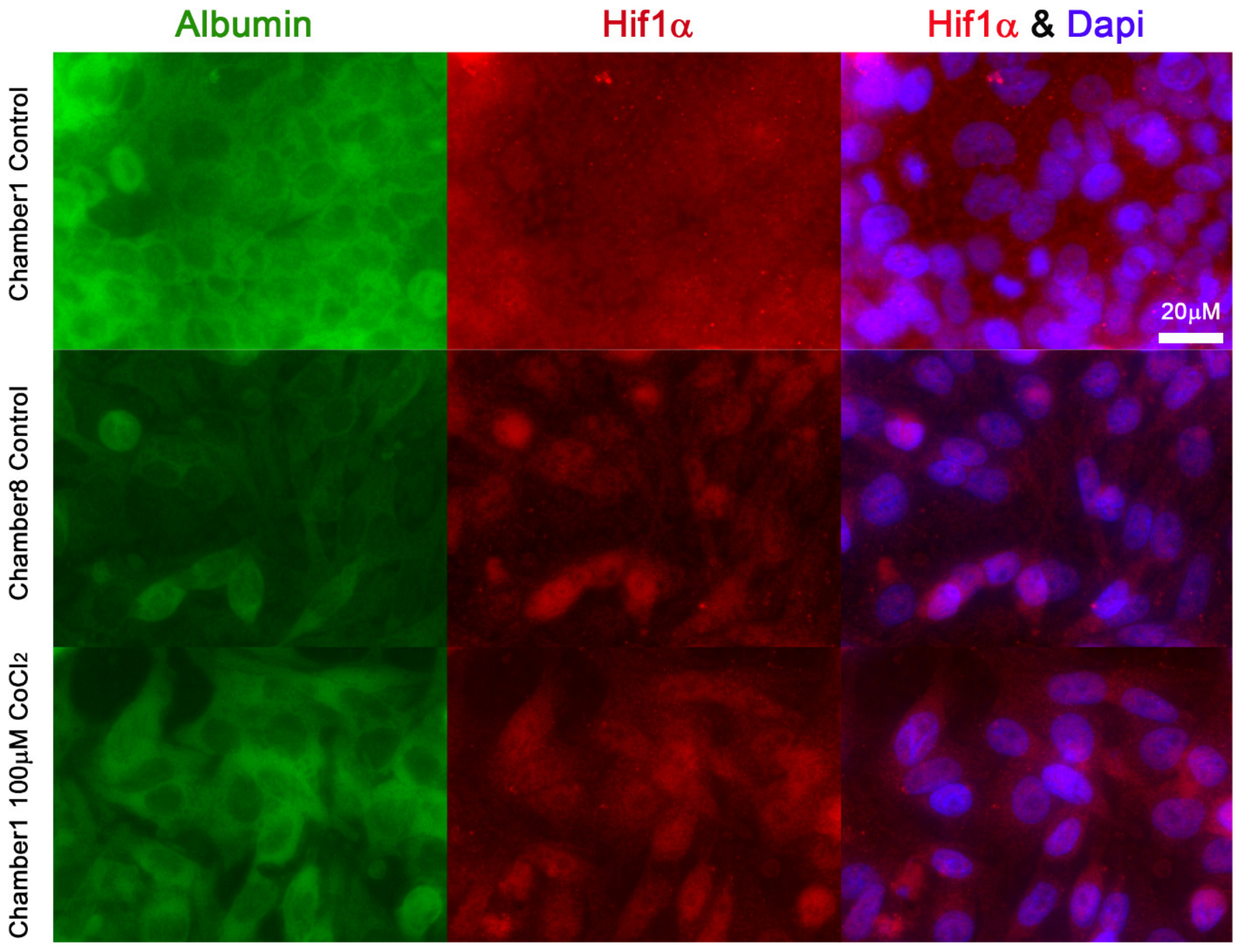

Supplementary Figure S5: Higher magnification of IF staining of albumin and Hif1α in chamber1, chamber8 (normal media) and chamber1 treated with $CoCl_2$. Albumin and Hif1α fluorescence in HepG2 cells in the connected MCs were imaged with a higher magnification. To visualize the Hif1α translocation difference from chamber1 to chamber8 and chamber1 treated with $CoCl_2$ in the right column an overlay of DAPI and Hif1α is shown.

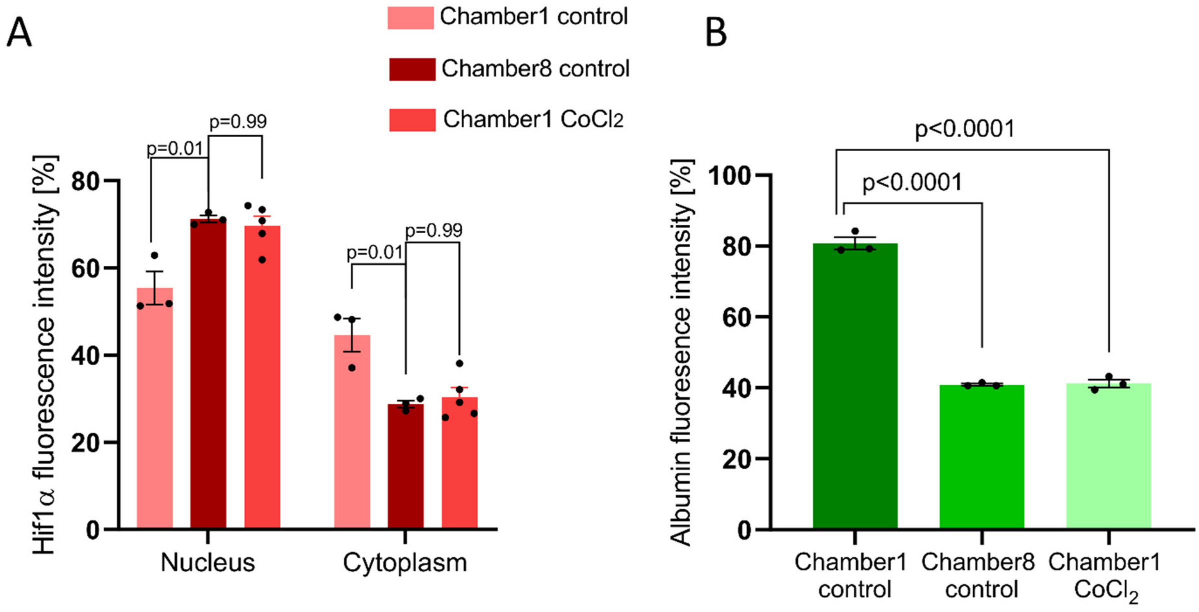

Supplementary Figure S6: Quantification of Hif1α and albumin fluorescence signals after $CoCl_2$ treatment. Relative fluorescence intensity of Hif1α (A) and albumin (B) in chamber1, chamber8 (normal media) and chamber1 treated with $CoCl_2$. The levels of Hif1α increase from chamber1 to chamber8 (p=0.01) and stay at the same level as chamber8 for chamber1 treated with $CoCl_2$ (A); the levels of albumin decrease from chamber1 to 8 (p<0.0001) and from chamber1 to chamber1 treated with $CoCl_2$ (B).
